# The Worst War of the World

**Published:** 1917-12-29

**Authors:** 


### THE WORST WAR OF THE WORLD.

### V. AN ENDURING COMMEMORATION.

### Our Old Army and Our First Defence.

So many momentous events have happened since 1914 that their united weight has clouded the memory of the parlous position to which Great Britain's powers of resistance and defence were reduced by politicians whose names and memory should one day be marked in history on a black list. This list should be preserved as a warning to the nation, and as an example of what may wisely be remembered as political folly so unpatriotic and dangerous to the people, as to rule out for all time from any active participation in the government of the British Nation and Empire, those responsible for it with their descendants. The politicians responsible for placing Great Britain in a position of vital peril, so real as to invite the destruction of the nation, and the reduction of its people to a position of dependence and servitude, would have secured by their unpatriotic folly and incompetence the ruin of a great people, had it not been for the splendid valour, self-sacrifice, and noble courage of the Old Army. No praise could be too high for them. They were our first and only defence. By the sacrifice of their lives, and the tenacity and self-effacement of their resolute resistance, they bought for us at a mortal price our only hope of ultimate safety. The Old Army were the men who in the time of peace made ready for inevitable war. They spent their best years in preparing themselves to serve their country. When the time of trial came they gave their lives with a chivalrous and never-faltering courage, fighting to hold the line unbroken, until their incredulous countrymen might, if God so willed, be trained to arms.

Of that Old Army, the first part (which had to bear the full weight of the invading forces of the German Army, organised and trained in numbers immeasurably exceeding those which their hosts had ever before reached in previous campaigns), amounted to so comparatively few that, 812,000 Germans were pitted against 320,000 men of the Old and French Army, when the German guns numbered 3,000 against some 1,200 guns of the Allies. These were the conditions. It can never be forgotten that Britain's troops amounted to only seven Divisions. They will be remembered for all time as the First Seven Divisions, who stood in the gap from the moment when fire was opened on August 23, until the final failure of the German attack at Ypres in November 1914. Of these seven, four were earliest in the field, and reached Mons on August 22, when they joined the Allied Forces under General Joffre.

The First and Second Divisions, forming the First Army Corps under Sir Douglas Haig, the Third and Fifth Divisions, forming the Second Army Corps under Sir Horace Smith Dorrien, the whole being under the command-in-chief of Field-Marshal Sir John French, arriving as they did on August 22, were engaged at dawn on August 23 by the opening of the fire of the German guns, and from 9 A.M. by their infantry, which attacked the British in close column. Our men shot like the bowmen of Cressy, and again and again their fire mowed down the enemy. Towards evening, under the ceaseless pressure of numbers, they were skilfully withdrawn in high spirits to a second position, but at 2 A.M came the unwelcome order to retire, and this retreat had to be continued until September 4 following, when the survivors had marched 150 miles in twelve days. Lord Ernest Hamilton has recorded how magnificently all branches of the Service worked throughout these trying conditions, when our men fought and retired without any possibility of adequate rest or sleep, until by pure grit and self-surrender September 6 came, when Sir John French, with an eager and reinforced army, was ready to join in the forward movement known as the Battle of the Marne. The further splendid and remarkable services rendered by the First Seven Divisions will be found recorded in Lord Ernest Hamilton's book,* which we commend for the attentive perusal of all classes of the British people.

It is not yet possible, adequately, to realise the achievement of our First Seven Divisions. At no time in our history have our national life and honour stood in such mortal danger; never in the history of war has an army so long resisted the attack of forces so huge and so disciplined, so heavily armed and so determinedly driven. This we shall know some day, when the tale is told in peace as part of the vast epic of war. It was felt, however, that already we could thank and commemorate those who saved us, for we could see them as they were and are. No men ever stood for Britain more truly than these; none ever represented more faithfully her quiet chivalry, her love of duty, her hatred of wrong, and none ever fulfilled more perfectly the ideals of a long and noble tradition. The Royal Albert Hall on December 15, 1917, contained an audience so vast that there was probably not a single unoccupied place, nor even a foot of standing room in the highest gallery—and they took part in the Choral Commemoration of the First Seven Divisions. The representatives of the survivors of this heroic army, numbering 700 officers, N.C.O.s, and men in khaki, were conspicuous in the boxes, in the galleries above, and in masses or small groups to the very top of the vast building. A notable feature were the regimental and other banners, made for the occasion, embroidered in many instances by women, who had travelled from the ends of the kingdom for the honour of putting in a few stitches. These were hung round the tiers,

* *The First Seven Divisions.* By Lord Ernest Hamilton. (London: Hurst and Blackett.)

☞ Previous articles appeared on July 28, August 11 and 18, and September 1.

**AN ENDURING COMMEMORATION—** *(continued).*

with the national flags, including a great White Ensign over the box occupied by Sir John Jellicoe and other naval officers. Their Majesties the King and Queen, Queen Alexandra, Princess Mary, Princess Alice (Countess of Athlone), and Princess Victoria, with their suites, occupied the Royal box. Field-Marshal Lord French, who commanded the "contemptible little Army"; Mr. Balfour, the Foreign Secretary; Lord Derby, the Secretary of State for War; Mr. Fisher, the Minister of Education; and Brigadier-Generals Turner and Gloster were noticeable in the assembly.

"The programme followed a scheme of ideas. The first item, Sir Edward Elgar's 'Cockaigne (in London Town),' stood for the light-hearted world before the war, Dr. Ralph Vaughan-Williams's song for chorus and orchestra, 'Towards the Unknown Region' (Whitman's 'Darest thou now, O Soul') spoke the 'challenge to the great adventure.' 'The cost' was figured by Mr. Howell's 'Elegy for Strings,' composed in memory of a friend killed in this war; 'the achievement,' by Mr. Arthur Somervell's Ode, 'To the Vanguard, 1914,' composed to the poem by Miss Beatrix Brice —'O little mighty Force that stood for England,' which first appeared on the front page of the *Times*. The solo in this was sung by Miss Lillian Stiles-Allen. Next came 'separation,' expressed by a motet for unaccompanied voices, which is one of the 'Songs of Farewell' written by Sir Hubert Parry for the years 1914-16; and Sir C. Villiers Stanford's song for bass solo and chorus (the vocalist being Mr. Plunket Greene), summed up, in the words of Sir Henry Newbolt's poem, 'Farewell,' the sacrifice, death, reunion, and immortality."

Dr. Hugh P. Allen, M.A., Mus.Doc. Oxon., was the conductor, a conductor who has been sadly wanted in recent years. He is a conductor with a whole-hearted mastery of his themes, possessed of the spirituality and power the absence of which have been grievously felt in recent years, when, for the want of the devotional spirit, the presentation of the greater oratorios has sunk to a level, which to many of us who regard these great works as ministering to the needs of our spiritual nature, has become a grievous outrage owing to the perfunctory and slovenly rendering, often hurried, always without soul and ever increasing in both defects. It is time a new conductor was found with the devotional spirit for oratorio music, or it must lose its hold on British people, to the great loss of the spiritually minded and devotional, who in large measure form the backbone of the nation. The music was well selected and beautiful. It had a marked effect upon all present, and the splendid voice of Mr. Plunket Greene was never heard to better advantage or was more welcome and so greatly appreciated by a vast audience of his countrymen. The programme by its arrangement and character held the audience throughout, and we must leave the able musical critic of the *Times* (to which we are greatly indebted), whose account we append, to do justice to it:—

### A CHORAL TRIUMPH.

### Music by English Composers.

"The music, all by living Englishmen, was wisely chosen and splendidly sung. After the Cockaigne Overture, which gained point by being taken at a brisk pace, came the manliest of English choral works—Vaughan-Williams's 'Towards the Unknown Region.' It goes deep below the show of things to the great thoughts they embody, and in doing that it set the right tone of feeling for the commemoration. Its breadth and stature are realised for the first time in this large space, which, with such an audience and occasion, seemed its natural home. That could not be said of Howell's 'Elegy' for strings which followed it, for it was with difficulty audible; but we should have been sorry to miss hearing a work which so worthily, and with such promise, upholds the true tradition.

"With Somervell's 'To the Vanguard,' now performed for the first time, the appeal was more direct and in so far less deep. The fault lay mainly with the poem, which has no impact, and contains no memorable line. Though the music does all that can be done with it, somehow it never sounded the note we were waiting to hear—that moment which lifts the particular to the universal, the transitory to the eternal. Miss Stiles-Allen made much of her short opportunity with a melodious soprano solo, and the tones of her voice, though tremulous, rang true.

"The finest effort of the choir, consisting of the Bach Choir, supported by the Royal Choral Society, was heard in Parry's 'There is an old belief.' The words offer a sound starting point, but the motet is a conspicuous instance of the power music has to illumine their message and drive home their meaning, as the setting sun and the incoming tide unite to transfigure a tall ship entering port. It was fitly followed by the song for bass and chorus, 'Farewell,' in which we did not know whether we were most deeply moved by Newbolt's words or Stanford's tones or Plunket Greene's voice, or by the time and place that made them what they were. Certainly Mr. Greene can seldom have had a more difficult task, or a task have shown more clearly what response the right man can make to it.

"If there was anything in which the programme erred it was in a certain uniformity; we wanted something like the Rondo of Beethoven's Funeral March sonata to cheer us up, and there would have been good military precedent for giving that turn to sad thoughts. Yet, perhaps, it was best as it was, now—and 'For all the Saints,' to Vaughan-Williams's tune, met the need in the best way. It would have been still better if the audience had noticed the intimation that their help was desired; some day they will sing it, and will realise what it has rescued them from.

"The orchestra owned some human imperfections, but atoned for them nobly. Mr. Darke's organ accompaniment was in good taste. Dr. Allen's conducting is a wonderful combination of energy, modesty, and good sense. No one would get the impression that he was 'doing it all'—yet

**AN ENDURING COMMEMORATION**—(*continued*).

he does a great deal, or, more truly, has done it before entering the room. He is there merely as one among others labouring for a cause he has at heart."

MR. BALFOUR AND LORD DERBY.

At the conclusion of the concert Mr. Balfour and Lord Derby ascended the platform, in front of which had been placed a laurel-wreathed replica of Mr. Richard Belt's bust of Lord Kitchener. The original was cast from captured cannon, and placed in the War Office. Mr. Balfour's personality and charm were made manifest in his manner and procedure as he shook hands with Dr. Hugh P. Allen and proceeded to read the verses from Ecclesiasticus xliv. 1-14, in a voice which carried through the great assembly and lingered in the memory. They begin, "Let us now praise famous men."

"There be of them, that have left a name behind them, that their praises might be reported.

"And some there be, which have no memorial; who are perished, as though they had never been; and are become as though they had never been born; and their children after them. . . .

"But these were merciful men, whose righteousness hath not been forgotten. . . .

"Their seed shall remain for ever, and their glory shall not be blotted oue."

Mr. Balfour's reading made a profound impression, and excited reverence. Lord Derby then read the Order of Battle of the First Seven Divisions. First came the names of Field-Marshal Lord French, the commanders of the four Army Corps, and the principal officers of the General Staff. The cheering of the men in khaki was stirring and eloquent. It justly distributed recognition of the great captains and the affection in which they were held by their troops and colleagues. Next the trumpeters and drummers of the Coldstream Guards, under Major Mackenzie Rogan, sounded the "Fall-in," followed from the extreme end of the hall by the sound of pipes and drums; then there entered some of the band of the Scots Guards, who marched straight up the centre playing the bagpipes as they came, and were received with loud cheers. Lord Derby, who was in splendid voice, resumed his reading, taking Division by Division, giving in each case the name of the commanding officer and the numbers and titles of the several units of Cavalry, Artillery, Engineers, and Infantry in each. Following the announcement of the Regulars came that of the Yeomanry and the Territorial battalions which had left the Homeland as reinforcements to the Expeditionary Force before November 23, 1914. It was a most striking, stirring, and, at times, amusing exhibition, owing to the cheering and regimental cries and humorous comments of the gallant 700 representatives of the Old Army.

SOME NOTABLE INCIDENTS.

It is noteworthy that the names of the Royal Army Medical Corps, the Army Service Corps, the Army Chaplains' Department, and Queen Alexandra's Imperial Military Nursing Service were received with rounds of cheering. This was striking testimony to the undoubted fact, that, in this war, the services of non-combatants, their valiant conduct and their courageous, faithful, and continuous output of the best they have to give, will make it ever memorable by the wonderful results the non-combatants, in conjunction with their combatant colleagues, have achieved. The singing of the hymn "For all the Saints" would have been much more effective, because it is so much better known, had it been to the heart-stirring tune used in cathedrals and churches. As it was, the moving music by Dr. Vaughan-Williams, divided into two sections, gained by the orchestral rendering of the words, but was less effective than the other tune would have been, because Dr. Williams' music was not known to a majority of the audience.

Then followed the National Anthem, three verses of which were sung, as they ought always to be sung on public occasions and in places of worship, with a go, unity and purposefulness, which gave to the words a meaning and reality which convention so often diminishes or destroys. No one who had the privilege of being present can ever forget this Commemoration at the Albert Hall. It was a memorable occasion, full of the deepest meaning, reality and feeling, which spoke to the 700 men in khaki with the voice of the whole Empire, and few, if any, present failed to be moved and stirred to the very depths. It demonstrated the nation's and Empire's gratitude and praise of famous men who are honoured in their generation, and whose glory shall not be blotted out.

A MESSAGE TO ABSENT COMRADES.

On the next day the following telegram was sent to the Commanders-in-Chief of the British Forces in France, Italy, Egypt, Salonica, and Mesopotamia:

"Those assembled here to-day in the Albert Hall, London, at a meeting honoured by the presence of their Majesties the King and Queen and Queen Alexandra to commemorate the heroic deeds of the First Seven Divisions, send their warmest greetings to all absent comrades and wish them good luck and a victorious homecoming."

